# Effect of machine learning methods on predicting NSCLC overall survival time based on Radiomics analysis

**DOI:** 10.1186/s13014-018-1140-9

**Published:** 2018-10-05

**Authors:** Wenzheng Sun, Mingyan Jiang, Jun Dang, Panchun Chang, Fang-Fang Yin

**Affiliations:** 1 School of Information Science and Engineering, Shandong University, Qingdao, Shandong 266237 People’s Republic of China; 2 Department of Radiation Oncology, Duke University Cancer Center, Durham, NC 27710 USA; 3 Department of Oncology, The First Affiliate Hospital of Chongqing Medical University, Chongqing, 400016 People’s Republic of China; 4 School of Electrical and Information Engineering, Qilu Institute of Technology, Jinan, Shandong 250200 People’s Republic of China

**Keywords:** Overall survival, Non-small cell lung cancer, Machine learning, Radiomics analysis

## Abstract

**Background:**

To investigate the effect of machine learning methods on predicting the Overall Survival (OS) for non-small cell lung cancer based on radiomics features analysis.

**Methods:**

A total of 339 radiomic features were extracted from the segmented tumor volumes of pretreatment computed tomography (CT) images. These radiomic features quantify the tumor phenotypic characteristics on the medical images using tumor shape and size, the intensity statistics and the textures. The performance of 5 feature selection methods and 8 machine learning methods were investigated for OS prediction. The predicted performance was evaluated with concordance index between predicted and true OS for the non-small cell lung cancer patients. The survival curves were evaluated by the Kaplan-Meier algorithm and compared by the log-rank tests.

**Results:**

The gradient boosting linear models based on Cox’s partial likelihood method using the concordance index feature selection method obtained the best performance (Concordance Index: 0.68, 95% Confidence Interval: 0.62~ 0.74).

**Conclusions:**

The preliminary results demonstrated that certain machine learning and radiomics analysis method could predict OS of non-small cell lung cancer accuracy.

**Electronic supplementary material:**

The online version of this article (10.1186/s13014-018-1140-9) contains supplementary material, which is available to authorized users.

## Background

Lung cancer is the leading cause of cancer-related deaths worldwide [1]. Lung cancer could be clinically divided into several groups: 1) the non-small cell lung cancer (NSCLC, 83.4%), 2) the small cell lung cancer (SCLC, 13.3%), 3) not otherwise specified lung cancer (NOS, 3.1%), 4) Sarcoma lung cancer (0.2%), and 5) other specified lung cancer (0.1%) [2]. The ability to predict clinical outcomes accurately is crucial for it allows clinicians to judge the most appropriate therapies for patients.

Radiomics analysis can extract a large number of imaging features quantitatively, which could offer a cost-effective and non-invasive approach for individual medicine [3–5]. Several studies have shown the predictive and diagnostic ability of radiomics features in different kinds of cancers using various medical imaging modalities, such as PET [6–8], MRI [9] and CT [4, 10, 11]. It is also demonstrated that the radiomic features are associated with the overall survival. Besides, these associations can be used to establish positive predictive models.

Machine-learning (ML) can be resumptively defined as the computational methods utilizing data/experience to obtain precise predictions [12]. The ML method can first learn laws from the data and then establish accuracy and efficiency prediction model based on these laws automatically. Moreover, an appropriate model is essential for the success use of radiomics. Hence, it is crucial to compare the performance of different ML models for clinical biomarkers based on radiomics analysis. Besides, appropriate feature selection methods should be applied first for the high-throughput radiomics features who may cause serious overfitting problems.

In this study, we investigated the effect of 8 ML and 5 feature selection methods on predicting OS for non-small cell lung cancer based on radiomics analysis. The effectiveness of ML and feature selection methods on the prediction of OS were evaluated utilizing the concordance index (CI) [6, 13–16].

## Methods

### Data acquisition

The data used in this study was obtained from the ‘NSCLC-Radiomics’ collection [4, 17, 18] in the Cancer Imaging Archive which was an open access resource [19]. All the NSCLC patients in this data set were treated at MAASTRO Clinic, the Netherlands. For each patient, manual region of interest (ROI), CT scans and survival time (including survival status) were available. All the ROIs in this data set were the 3D volume of the gross tumor volume (GTV) delineated by a radiation oncologist.

### Prediction process

**Fig. 1:**
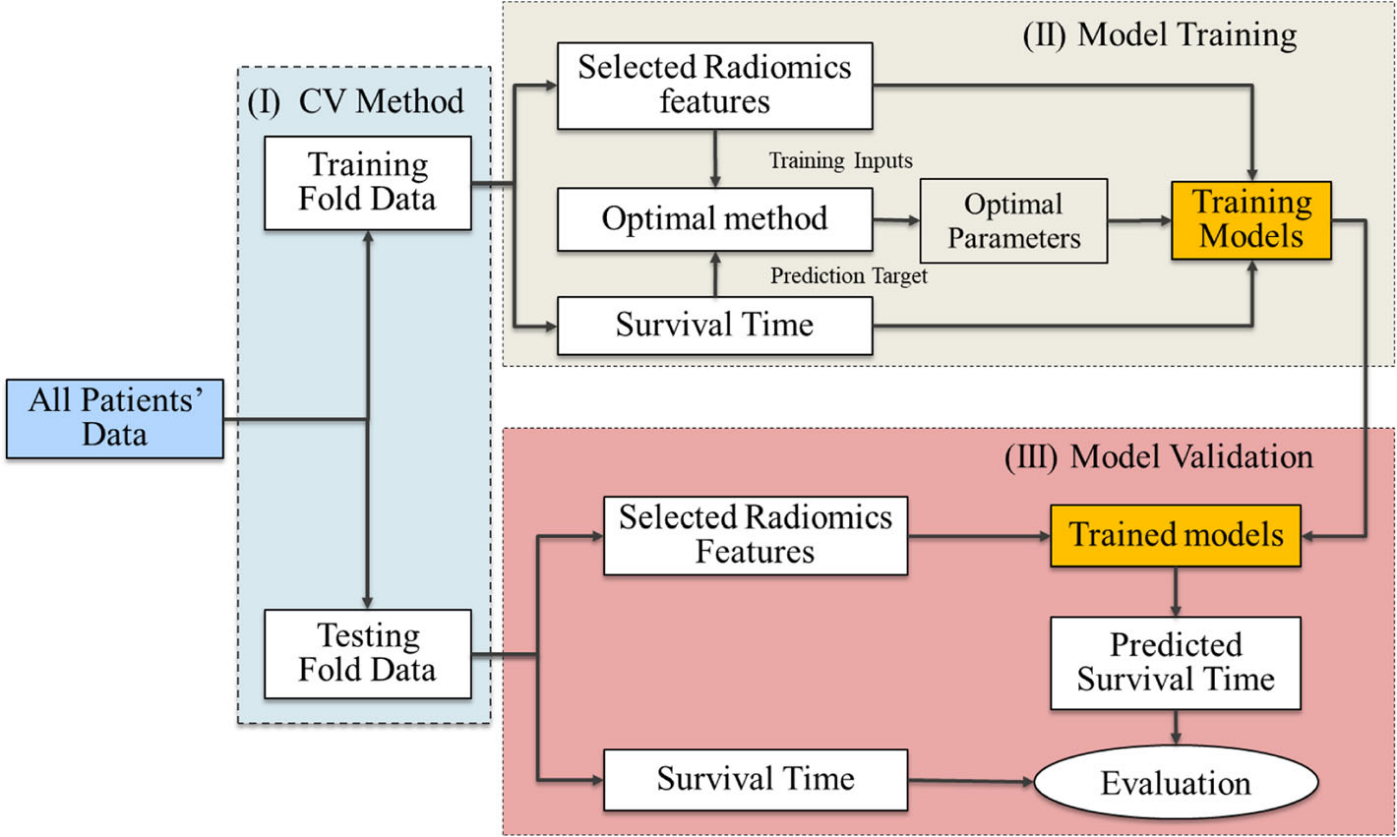
The flow chart of predicted process for each ML method. (I) Dividing total data into three folds using the cross validation method. (II) Training each ML model using the selected radiomics features of the training fold. (III) Validating the prediction performance of each ML model on the validation fold

The flow chart of the prediction process [20, 21] for all the ML methods in this study was outlined in Fig. 1. The performance of each ML and feature selection methods for the 283 NSCLC patients were evaluated using the cross-validation (CV) method (3-CV in this study). For each CV process, the total patients were divided into three folds, in which two folds (training fold) for training the machine learning model and the third (validation fold) for evaluating the model.

For each training fold, the training algorithm required both the training inputs (for prediction) and the prediction targets (for validation) data. The training inputs referred to the selected radiomics features, while the prediction targets referred to the OS of the patients. The radiomics features were first extracted from the images and then selected (dimension reduction) using the filter based feature selection methods to reduce the risk of overfitting. Finally, the selected features would be used to optimize and train all the ML models. In this study, the Bayesian optimization method was applied to determine the optimal parameters [22].

For each validation fold, the corresponding selected radiomics features were first extracted from the images and then transferred into the trained model. Finally, the prediction OS would be used to evaluate the goodness of each model.

### Image pre-processing and Radiomics features extraction

**Fig. 2:**
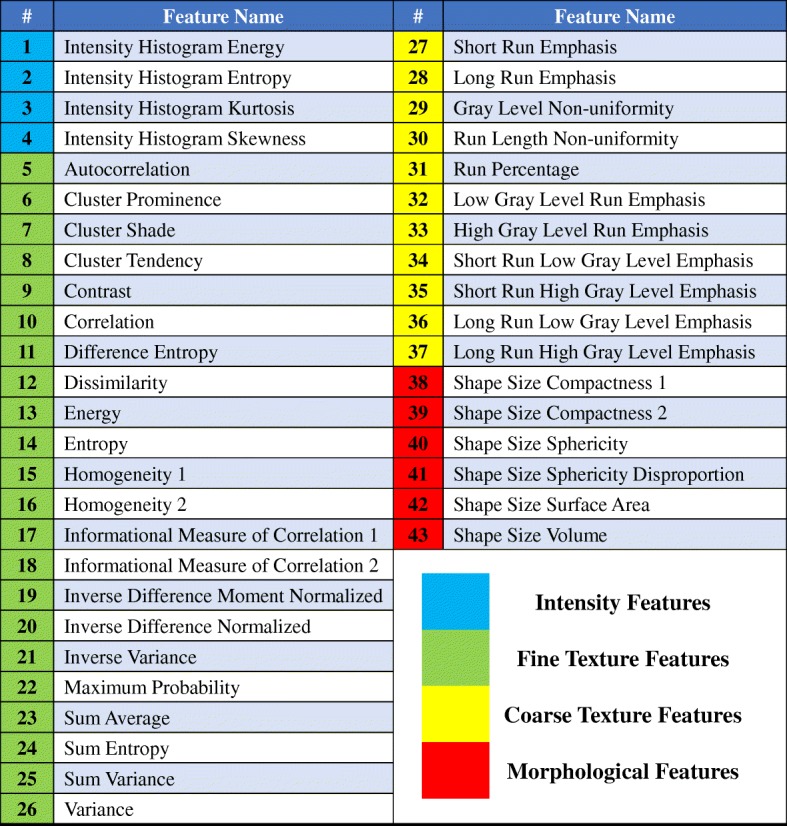
Radiomics features used in this study. The definitions of radiomics features could be found in the IBSI document [26]. (I) Intensity features (1–4): 3.4.19, 3.4.18, 3.3.4 and 3.3.3 sections; (II) Fine texture features (5–26): 3.6.20, 3.6.23, 3.6.22, 3.6.21, 3.6.12, 3.6.19, 3.6.7, 3.6.5, 3.6.11, 3.6.4, 3.6.14, 3.6.16, 3.6.24, 3.6.25, 3.6.17, 3.6.15, 3.6.18, 3.6.1, 3.6.8, 3.6.10, 3.6.9 and 3.6.3 sections; (III) Coarse texture features (27–37): 3.7.1, 3.7.2, 3.7.9, 3.7.11, 3.7.13 and 3.7.3–3.7.8 sections; (IV) Morphological feature: 3.1.5, 3.1.6, 3.1.8, 3.1.7, 3.1.3 and 3.1.1 sections

Prior to extracting the radiomics features, we fixed the bin number (32 bins) of all the pre-treatment CT scans to discretize the image intensities. It should be noted that the original voxels for the images were used in this study. Then, the radiomics features were automatically extracted from the GTV region of the CT images by our in-house developed radiomics image analysis software and the Wavelet toolbox based on the Matlab R2017a (The Mathworks, Natick, MA). Total 43 unique quantitative features in 4 categories (Fig. 2) were extracted:

1) Intensity features: to describe the shape characteristics of the CT volume’s gray-level intensity histogram, i.e., a probability density function (PDF) of gray-level distribution.

2) Fine texture features: to describe the high-resolution heterogeneity in the ROI. These features were derived from the ROI’s Gray-Level Co-Occurrence Matrix (GLCOM), a joint PDF that measures the frequency of co-occurring adjacent voxel pairs having the same grayscale intensity at a given direction [23].

3) Coarse texture features: to describe the low-resolution heterogeneity in the ROI. These features were calculated from the ROI’s Gray-Level Run Length Matrix (GLRLM), a joint PDF that measures the size of a set of consecutive voxels with the same grayscale intensity at a given direction [24].

4) Morphological features: to describe the morphological characteristics of the ROI [25].

Here, the first category and the following two (second and third) categories required the intensity histogram and textural image processing steps, respectively. Both the above two image processing steps and the 43 radiomics features used in this study matched benchmarks of the Image Biomarker Standardization Initiative (IBSI) [26].

Moreover, these radiomics features were also extracted from different wavelet decompositions of the original CT image by a three levels wavelet transformation [27, 28]. However, the morphological features weren’t extracted from the images with the wavelet decompositions for the wavelet transformation didn’t have effect on these features. Hence in total, 339 features were extracted for each patient in this study.

### Features selection and machine learning methods

Pearson’s (PCC) [29], Kendall’s (KCC), [30] Spearman’s linear correlation coefficient (SCC) [31], Mutual information (MI) [32] and CI [15] were used as the filter based feature selection methods to reduce the dimensions of radiomics features in this study. In order to make sure the reliability of the selected features, we repeated each feature selection process 100 times using the bootstrap samples of each training fold and recorded the selected feature subset each time. Then, we selected the most frequently selected radiomics features as the final features which were used to train the ML models [6]. In this study, the first four feature selected methods (PCC, KCC, SCC and MI) were implemented using the Matlab R2017a and the following one method (CI) was implemented using the R software 3.5.1. All the feature selection methods would be performed on each training fold.

The effect of 8 ML methods were investigated in this study, including: Cox proportional hazards model (Cox) [33], gradient boosting linear models based on Cox’s partial likelihood (GB-Cox) [34], gradient boosting linear models based on CI’s partial likelihood (GB-Cindex) [34], Cox model by likelihood based boosting (CoxBooxt) [35], bagging survival tree (BST) [36], random forests for survival model (RFS) [37], survival regression model (SR) [38] and support vector regression for censored data model (SVCR) [39, 40]. All the machine learning methods were implemented on each training fold using the R software 3.5.1. The specifics of the packages for each feature selection and ML method were showed in the Table 1. Besides, the descriptions of each feature selection and ML method could be found in the Additional file 1: Supplementary A and B, respectively.

**Table 1:** The specifics of the packages for each feature selection and machine learning method

Methods	Software	Packages	Website Links
PCC	SML toolbox	corr	https://ww2.mathworks.cn/help/stats/corr.html
KCC			
SCC			
MI	MIToolbox	mi	https://github.com/Craigacp/MIToolbox
CI	Hisc	rcorr.cens	https://github.com/harrelfe/Hmisc
Cox	survival	coxph	https://github.com/therneau/survival
GB-Cox	mboost	mboost	https://github.com/boost-R/mboost
GB-Cindex	mboost	mboost	https://github.com/boost-R/mboost
CoxBoost	CoxBoost	CoxBoost	https://github.com/binderh/CoxBoost
BST	ipred	bagging	https://github.com/cran/ipred
RFS	randomForestSRC	rfsrc	https://github.com/kogalur/randomForestSRC
SR	survival	survreg	https://github.com/therneau/survival
SVCR	survivalsvm	survivalsvm	https://git-hub.com/imbs-hl/survivalsvm

*SML* statistics and machine learning

### Parameters tuning

For each ML method, the parameters were selected from the combination of parameters that produced the best performance using the three-fold CV on each training fold. Similar procedures were implemented in Brungard et al. [41] and Heung B et al [42].

The range of parameters used in this study was showed in Table 1. The GB-Cox, GB-Cindex, SVCR and SR methods just required one parameter to tune while the Cox method did not require parameterization. The complex models, such as the BTS and RFS, were time consuming for tuning parameters. The parameters from all of these models, such as the average terminal node size of forest and the number of trees for the RFS model, the minimum number of observations that must exist in a node (Minsplit) and the number of trees for BST, made up a large range of parameter permutation and combination choices. It should be noted that the feature number selected by the feature selection methods were also used as a tuning parameter (range [3, 29]) for all the ML methods.

### Evaluation methods

CI with confidence interval (CFI) based on bootstrapping technique (the number of bootstrap samples was 2000 in this study) was used to assess the performance of difference ML methods on the merged validation fold (merged all the three validation folds). The percentage of CFI was 95% in this study. A nonparametric analytical approach method proposed by Kang L et al. [43] and the z-score test method were used to compare the significance between pairs of machine learning algorithms for each validation fold. Besides, the survival curves were evaluated by the Kaplan-Meier algorithm and compared by the log-rank tests [44] for each validation fold.

## Results

**Fig. 3:**
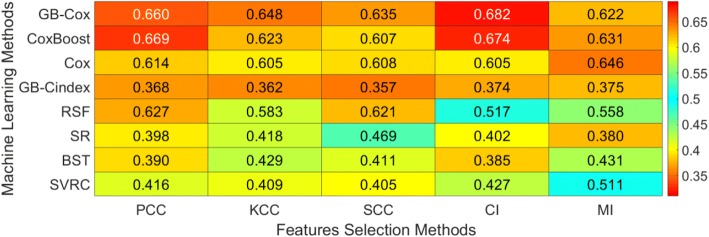
The performance of feature selection and machine learning methods on the merged validation fold

Figure 3 depicted the performance of ML (in rows) and feature selection methods (in columns) on the merged validation fold. Besides, the maximum CI with confidence interval for each ML method on the merged validation fold was showed in Table 2. The GB-Cox method using the CI feature selection method obtained the best performance (CI: 0.682, 95% CFI: [0.620, 0.744]). However, the CoxBoost method using CI feature selection method also obtained a favorable performance (CI: 0.674, 95% CFI: [0.615, 0.731]). We found only the above mentioned two prediction method’s CIs were close. Hence, we just calculated the *p*-value using the z-test between the above two methods. The p-value of CI between these two methods was 0.5, indicating that the difference of prediction performance between these two methods wasn’t significant. The values selected for the hyper-parameters mentioned in Table 3, as well as the number of selected features on each validation fold could be found in the Additional file 1: Supplementary C.

**Table 2:** Maximum CI with confidence interval for each machine learning method on the merged validation fold

Methods	FS	Maximum CI	CFI of Maximum CI
GB-Cox	CI	0.682	[0.620, 0.744]
CoxBoost	CI	0.674	[0.615, 0.731]
Cox	MI	0.646	[0.578, 0.714]
GB-Cindex	SCC	0.357	[0.290, 0.423]
RFS	PCC	0.627	[0.558, 0.695]
SR	MI	0.380	[0.310, 0.452]
BST	SCC	0.385	[0.318, 0.450]
SVCR	KCC	0.405	[0.341, 0.470]

*FS* feature selection method

**Table 3:** The range of parameter tuning

Methods	Parameters	Range of Parameters
Cox		
GB-Cox	Number of boosting steps	[1, 500]
GB-Cindex	Number of boosting steps	[1, 500]
Coxboost	Number of boosting steps	[1, 500]
BST	Minsplit	[1, 10]
	Number of trees	[1, 500]
RFS	Average terminal node size of forest	[1, 10]
	Number of trees	[1, 500]
SR	Assumed distribution	Weibull, Gaussian, Exponential
SVCR	Parameter of regularization	[0.01, 1]

**Fig. 4:**
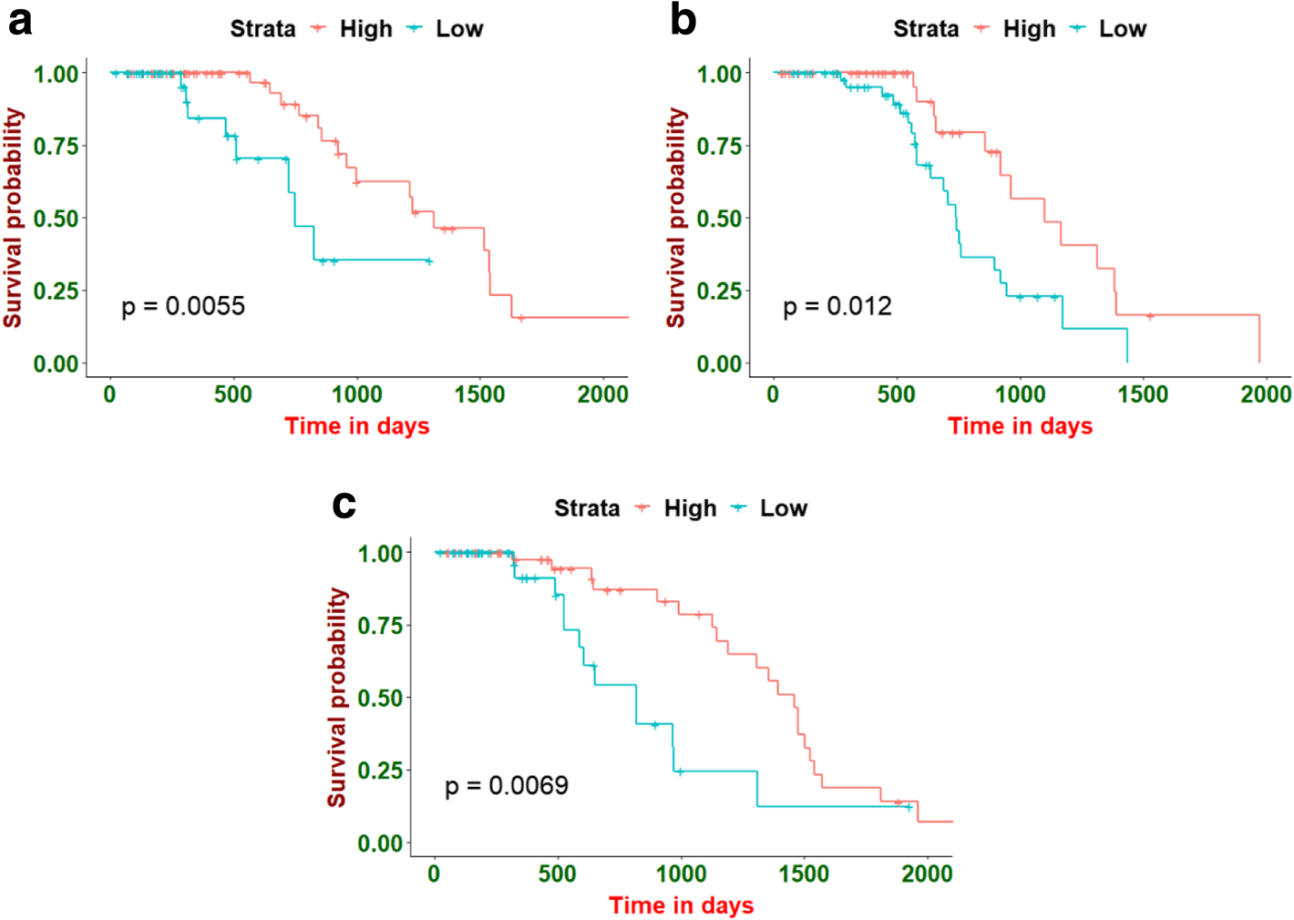
Examples of the Kaplan-Meier evaluations. All the NSCLC patients on each validation fold were stratified into low- and high- risk groups based on the cut-off values determined by the corresponding training fold. Here, (**a**), (**b**) and (**c**) presented the Kaplan-Meier curve of the three CV validation folds, respectively

Patients on each validation fold were divided into two groups (low- and high- risk group) based on the predicted risk of each radiomics model at the cut-off value. The cut-off value utilized for stratification was the median of each training fold which would be applied to the corresponding validation fold unchanged. Then, the Kaplan-Meier and log-rank tests methods were used to evaluate and compare the survival curves for each validation fold, respectively. Among all the ML methods, the GB-Cox method with the CI feature selection method obtained the best stratified result on the 3 CV folds (Fig. 4). Besides, the *p*-value of the CoxBoost method with the PCC feature selection method was also significant for each validation fold. The heatmap of *p*-values on each validation fold for all the ML methods was showed in the Additional file 1: Supplementary D.

## Discussion

Several previous studies have compared the prediction performance of the ML models based on the radiomics analysis. Parmar C et al. [11] identified that three classifiers, included Bayesian, random forest (RF) and nearest neighbor, showed high OS prediction performance for the head and neck squamous cell carcinoma (HNSCC). Parmar C et al. [17] also evaluated the effect of ML models (classifiers) on the OS prediction for NSCLC patients and found that the random forest method with Wilcoxon test feature selection method obtained the highest prediction performance. However, the outcome of interest in these two studies explored by Parmar C et al. was transformed into a dichotomized endpoint. This may lead to the bias of prediction accuracy [13]. Hence, Leger S et al. [13] assessed the prediction performance (OS and loco-regional tumor control) of ML models which could dealt with continuous time-to-event data for HNSCC. His study found that the random forest using maximally selected rank statistics and the model based on boosting trees using CI methods with Spearman feature selection method got the best prediction performance for the loco-regional tumor control. Besides, the survival regression model based on the Weibull distribution, the GB-Cox and the GB-Cindex methods with the random feature selection method achieved the highest prediction performance for the OS. In this study, the effect of 8 ML models and five feature selection methods based on radiomics feature analysis were investigated to predict the time-to-event data (OS) of non-small cell lung cancer. In general, the GB-Cox method obtained the best predictive performance in the systematic evaluation on the merged validation fold. However, the CoxBoost methods with certain feature selection method also showed comparable positive performance compared with the GB-Cox method. Hence, we thought a wide range of ML methods have the potential to be effective radiomics analysis tools. Besides, a significant difference for OS prediction on each validation fold was found between the low- and high- risk groups using the GB-Cox and CoxBoost methods, which showed the clinical potential of ML methods on the OS prediction.

As shown in Fig. 3, almost all of the ML methods using the KCC feature selection method didn’t obtain a positive result. This indicated that the feature selection method was also important for the performance of OS prediction. Sometimes, the effect of feature selection methods was even more obvious than the ML models. A large panel of feature selection methods had been used for data mining of high-throughput problems [45, 46]. In general, the feature selection methods would be divided into three categories: the filter based, the wrapper based and the embedded methods. In this study, we only investigated five different filter based methods because this kind of methods were not only less prone to overfitting but also more efficient in computation than other two methods [45, 46]. Moreover, the filter based methods were more independent than the wrapper and embedded methods, which could increase the fairness of ML methods comparison.

Some previous studies [4, 5] have shown the potential clinical utility of the prognostic models based on radiomics analysis. This study could be a crucial supplementary reference for the use of prognostic models based on radiomics analysis because we compared a large number of machine-learning methods for the OS prediction of the NSCLC cancer. Such a comparison would be helpful in the selection of the optimal ML methods for OS prediction based on radiomics analysis.

## Conclusion

The preliminary results demonstrated that certain machine learning and radiomics analysis method could predict OS of non-small cell lung cancer accuracy.

## Additional file

Additional file 1: Supplementary A: Feature selection methods. Supplementary B: Machine learning methods. Supplementary C: The values selected for the hyper-parameters on each validation fold. Supplementary D: *P*-values of the log-rank test for all the feature selection and ML methods on each validation fold. (PDF 625 kb)

## Abbreviations

BST: Bagging survival tree
CFI: Confidence interval
CI: Concordance index
Cox: Cox proportional hazards model
CoxBoost: Cox model by likelihood based boosting
CT: Computed tomography
CV: Cross-validation
GB-Cindex: gradient boosting linear models based on concordance index
GB-GB-Cox: gradient boosting linear models based on Cox’s partial likelihood
GLCOM: Gray-level co-occurrence matrix
GLRLM: Gray-level run length matrix
GTV: Gross tumor volume
HNSCC: head and neck squamous cell carcinoma
KCC: Kendall’s correlation coefficient
MI: Mutual information
ML: Machine-learning
NSCLC: Non-small cell lung cancer
OS: Overall survival
PCC: Pearson’s correlation coefficient
PDF: Probability density function
RFS: Random forests for survival model
ROI: Region of interest
SCC: Spearman’ linear correlation coefficient
SCLC: Small cell lung cancer
SR: Survival regression model
SVCR: Support vector regression for censored data model
