# Supplementary material for: Effect of machine learning methods on predicting NSCLC overall survival time based on Radiomics analysis
Source: Radiat Oncol. 2018 Oct 5;13:197. doi: 10.1186/s13014-018-1140-9 (PMC6173915; doi:10.1186/s13014-018-1140-9)
Supplement: Supplementary file 1 — Supplementary A: Feature selection methods. Supplementary B: Machine learning methods. Supplementary C: The values selected for the hyper-parameters on each validation fold. Supplementary D: P-values of the log-rank test for all the feature selection and ML methods on each validation fold. (PDF 625 kb) [file 13014_2018_1140_MOESM1_ESM.pdf]

Supplementary A: Feature selection methods

Five methods, including PCC, KCC, SCC, MI and CI, are used in this study to select the useful features. For the first four algorithms (PCC, KCC, SCC and MI), only those cases for which the event occurred are considered. On the contrary, all the cases are used to select the useful features for the CI feature selection method.

◆ Correlation coefficient:

The first three correlation coefficient algorithms are non-parametric methods used for measuring the linear dependency between two variables.

The Pearson correlation coefficient (PCC) between the feature *x* and the times y is defined as following:

$$Corr_P = \frac{\sum_{i=1}^{n}(x_i - \overline{x})(y_i - \overline{y})}{\sqrt{\sum_{i=1}^{n}(x_i - \overline{x})^2 \sum_{i=1}^{n}(y_i - \overline{y})^2}} \tag{1}$$

The Kendall correlation coefficient (KCC) between the feature *x* and the times y is defined as following:

$$Corr_K = \frac{2K}{n(n-1)} \tag{2a}$$

$$K = \sum_{i=1}^{n}\sum_{i=1}^{n} \xi^*(x_i, x_j, y_i, y_j) \tag{2b}$$

$$\xi^*(x_i, x_j, y_i, y_j) = \begin{cases} 1 & if \quad (x_i - x_j)(y_i - y_j) > 0 \\ 0 & if \quad (x_i - x_j)(y_i - y_j) = 0 \\ -1 & if \quad (x_i - x_j)(y_i - y_j) < 0 \end{cases} \tag{2c}$$

The Spearman correlation coefficient (SCC) is equivalent to PCC applied to the rankings of the columns *X* and *Y*. when all the ranks in each column are distinct, the equation simplifies to:

$$Corr_S = 1 - \frac{\sum 6d^2}{n(n^2-1)} \tag{3}$$

Here, *d* and *n* are the difference between the ranks of the two columns and length of

each column, respectively.

◆ Mutual information (MI):

MI is a method applied to measure the mutual dependence between the two variables. The equation is defined as following:

$$MI_S = \sum_{y\in Y}\sum_{x\in X} p(x,y)\log(\frac{p(x,y)}{p(x)p(y)}) \quad (4)$$

$p(x),\ p(y)$ are the marginal probability distribution functions of X and Y, respectively. $p(x,y)$ represents the joint probability function of X and Y.

◆ Concordance Index (C-index):

The C-index utilizes the relative risk of an event instead of the absolute survival times to evaluate a model in survival analysis. The main steps include:

1) Create all pairs of observed responses.
2) For all valid observed response pairs, i.e., $x_1>x_2$, test whether the corresponding prediction responses are concordant, i.e., $y_1>y_2$. If so, add 1 to the running sum (*Sum*). If $y_1=y_2$, add 0.5 to the *Sum*. Then, count the number (*n*) of valid response pairs.
3) Divide *Sum* by *n*.

Supplementary B: Machine learning methods

● Cox proportional hazards model (Cox):

The hazard function of this model is formulated as following:

$$\lambda(t|\mathrm{X}) = \lambda_0(t)e^{\beta X} \quad (5)$$

Here, the λ_0 and β are the baseline hazard function and regression coefficients, respectively. The β can be estimated using the partial log-likelihood:

$$LL(\beta) = \sum_{i=1}^{n} \delta_i(\beta X - \log(\sum_{k:k\geq t_i} \exp(\beta X_k)) \quad (6)$$

● Gradient boosting linear model based on CI and Cox (GB-Cindex and GB-Cox):

The target of the gradient boosting linear models is to establish a function to find $y = f^*(Y|X,\lambda)$ from data X and Y. The functional mapping is learned by minimizing the loss function ϕ of the empirical risk:

$$f^*(Y|X,\lambda) = \min_f \sum_{i=1}^{n} \phi\big(Y, f(x,\lambda)\big) \tag{7}$$

Here, f is the base-learner.

The gradient boosting method calculates the negative gradient of the loss function at each iteration (m=1, …, $mstop$) and evaluates it at $f^{m-1}(X,\lambda), i = 1, \dots, n$. This yields the negative gradient vector for each base learner. The negative gradient vector is defined as following:

$$u^m := -\frac{\partial \phi}{\partial f}\big(Y, f^{m-1}(X,\lambda)\big) \qquad t = 1,2,\dots,n \tag{8}$$

Typically, one base learner is utilized for each covariate and result in prediction values. Then, the $\hat{u}^m$ is set equal to the fitted values from the corresponding best base learner. Finally, the current estimate is updated by setting $\hat{f}^m = \hat{f}^{m-1} + v\hat{u}^m$. Here, v in range (0,1] is the length factor. The different of GB-Cindex and GB-Cox is the loss function ϕ. That is the GB-Cindex method used the concordance index (CI) while GB-Cox used the Cox's partial likelihood (Cox) as the lost function to be optimized.

- Cox model by likelihood based boosting (CoxBoost):

This model is used to fit a Cox proportional hazards model by component wise likelihood based boosting. In contrast with the GB-Cox, the CoxBoost model is not based on the gradients of loss functions but uses the offset-based boosting proposed by Tutz (Tutz, 2007) for evaluating the Cox proportional hazards models. In each boosting step, the previous boosting steps are contained as an offset in the penalized partial likelihood evaluation, which is applied for obtaining an update for one single parameter in every boosting step. The main complexity parameter of this model is the number of boosting steps (stepno). The instruction of the R package "Coxboot" also recommended to optimize this parameter by cross validation or other hyper-parameter setting methods.

The instruction also shows that the penalty value parameter (penalty) can be selected rather coarsely.

- Bagging survival tree model (BST):

Bagging is one of the most common methods which is typically used to reduce the variance of the base learners. In the bagging survival tree model, the survival function can be obtained by averaging the predictions calculated from a single survival tree (Hothorn et al. 2004). There are mainly 3 steps in the BST method: 1). Implement m bootstrap samples for the given data. 2). For each bootstrap sample, establish a survival tree. Then ensure that, for all the terminal nodes, the number of events is greater than or equal to the threshold. 3). Compute the bootstrap survival function by averaging the predictions of the leaf nodes. For each leaf node, the Kaplan-Meier estimator is used to estimate the survival function.

- Random forests for survival model (RFS):

The Random forests for survival model is an extension of Breiman's random forest algorithm (Breiman, 2001) for survival data. The basic aim is to draw ntree bootstrap samples from the training cohort. For each sample, a survival tree is trained. For each node of the tree, mtry (p/3 in this study) variables (features) are selected randomly as splitting candidates. Here, p is the number of features. For each splitting candidate, the maximum of split points (nSplit) are selected randomly among the possible split points. The logrank splitting is used as the splitting rule criteria for survival data. The process of selecting splitting candidates and split points will continue to repeat until the terminal nodes contains no less than nodeSize unique events. Based on the resulting tree ensemble, cumulative hazard is estimated by integrating all the information of the ntree trees. It should be noted that the instruction of the R package "randomForestSRC" recommended to optimize the nodeSize by multiple experiments (Ishwaran, 2018).

- Survival regression model (SR):

This model is a fully-parametric model which can offer different survival functions, such as Weibull, Gaussian and so on. For example, the Weibull probability density

function can be as defined following:

$$f(t) = \frac{\lambda t^{\lambda-1}}{\alpha^{\lambda}} \cdot e^{-\left(\frac{t}{\alpha}\right)^{\lambda}} = h(t) \cdot S(t) \tag{9}$$

Here, *S(t), h(t),* α, λ, are survival function, hazard function, scale and shape of the Weibull distribution. Then the hazard function is formulated:

$$h(t, X, \beta, \lambda) = \lambda e^{-\lambda X\beta} t^{\lambda-1} \tag{10}$$

Where $\lambda = 1/\sigma$ is defined.

- Support vector regression for censored data model (SVCR):

The core idea of method is to find a function which could estimate observed survival times (continuous outcome y_i) using covariates x_i based on the conventional support vector regression (SVR) (Vapnik, 1998). The SVCR model can be formulated as following:

$$\min_{\psi,b,\epsilon,\epsilon^*} \frac{1}{2}\|\psi\|^2 + \gamma \sum_{i=1}^{n} (\epsilon_i + \epsilon_i^*),$$

$$subject\ to \begin{cases} \langle \psi, F(x_i) \rangle + b \geq y_i - \epsilon_i & \forall\, i = 1, \dots, n \\ -\delta_i(\langle \psi, F(x_i) \rangle + b) \geq -\delta_i(y_i) - \epsilon_i^* & \forall\, i = 1, \dots, n \\ \epsilon_i \geq 0 & \forall\, i = 1, \dots, n \\ \epsilon_i^* \geq 0 & \forall\, i = 1, \dots, n \end{cases} \tag{11}$$

Here, δ, F, γ, ϵ, ϵ^* are the censoring indicator, the function that translates the observed covariates to the feature space, the strict regularization constant and the slack variables allowing for the errors in the training data predictions.

Supplementary C: The values selected for the hyper-parameters on each validation fold.

The following table and figure show the values selected for the hyper-parameters mentioned in table 2 and the number of selected features, respectively. Here, the CV in the table and figure represents the number of the validation fold.

		GB-Cox	CoxBoost	GB-index	RFS		SR	BST		SVCR
FS	CV	NBS	NBS	NBS	TN	NT	AD	MS	NT	PR
PCC	1	107	33	480	7	307	II	5	201	1
	2	1	192	500	9	240	II	1	401	0.96
	3	196	130	239	8	442	II	4	43	0.88
KCC	1	17	442	422	10	239	II	9	357	1
	2	46	500	500	10	433	II	9	41	1
	3	156	46	345	6	440	II	3	170	0.86
SCC	1	257	432	425	10	239	I	9	357	1
	2	1	13	357	10	265	III	4	153	1
	3	81	1	500	9	82	II	9	500	1
CI	1	500	239	500	10	500	II	3	500	1
	2	361	167	500	1	220	II	10	224	1
	3	124	130	43	10	500	II	10	355	1
MI	1	102	147	480	2	497	II	5	201	0.01
	2	500	130	463	1	1	II	1	415	0.85
	3	81	55	497	10	367	II	4	43	1

FS: Feature selection method.
I: Weibull, II: Gaussian, III: Exponential
NBS: number of boosting steps, TN: average terminal node size of forest, NT: number of trees, AD: assumed distribution, MS: minimum number of observations that must exist in a node, PR: parameter of regularization.

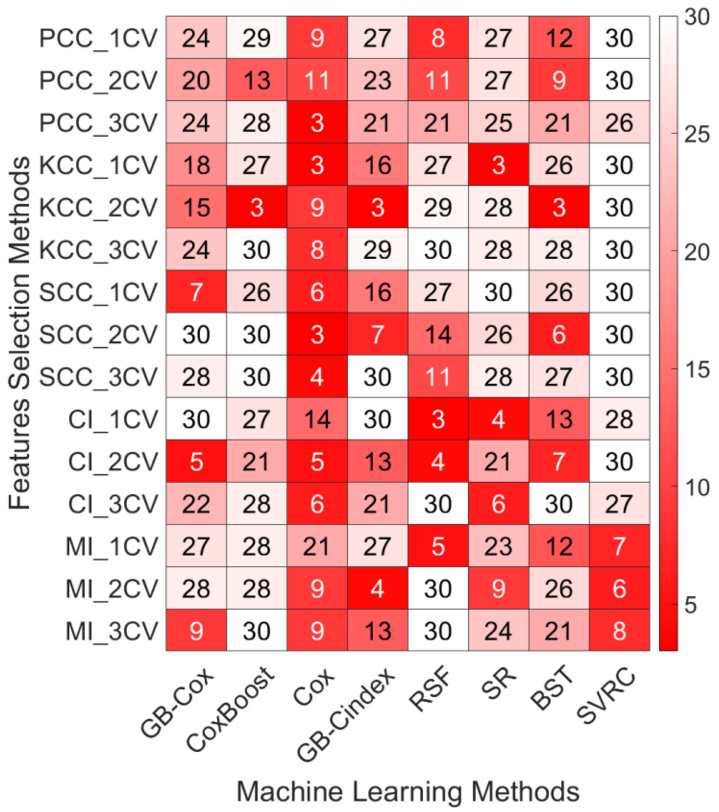

Supplementary D: P-values of the log-rank test for all the feature selection and ML methods on each validation fold

The following figure shows p-values of the log-rank test for all the feature selection and ML methods on each validation fold.

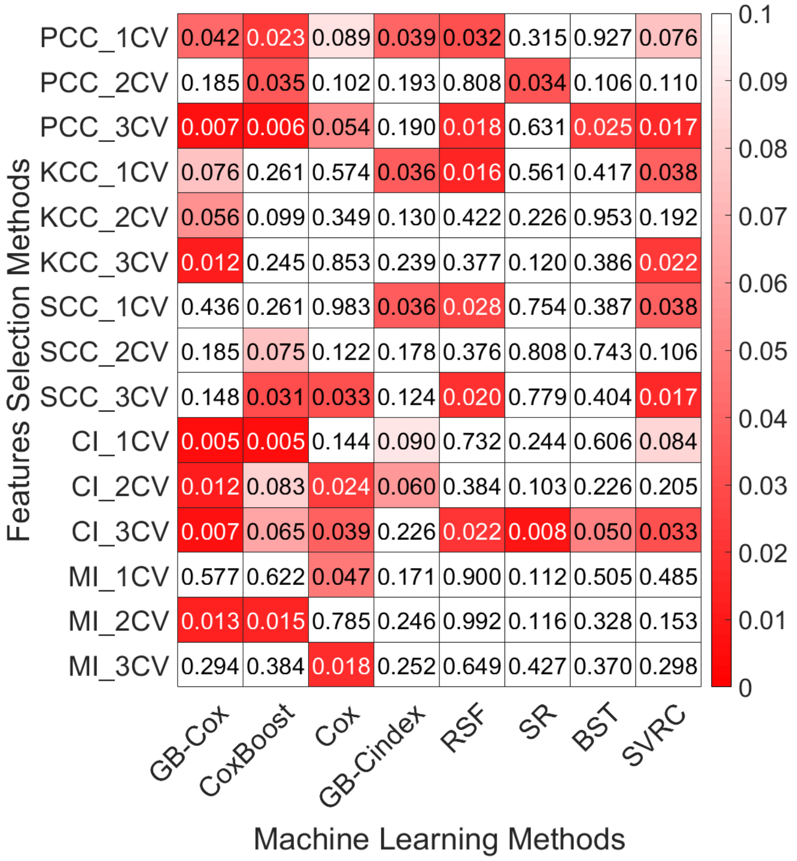

References:

1. Tutz G and Binder H, Boosting ridge regression. Computational Statistics \& Data Analysis, 2007; 51(12): 6044-6059.
2. Hothorn T, Buehlmann P, el al. Package “mboost” Title Model-Based Boosting 2018.
3. Breiman L, Random forests. Machine Learning. 2001; 45(1): 5–32.
4. Ishwaran H and Kogalur UB. Package “randomForestSRC” Title Random Forests for Survival, Regression, and Classification (RF-SRC) 2018.
5. Vapnik VN. Statistical Learning Theory. John Wiley & Sons, New York: 1998.
